# Posterior approach to correct for focal plane offsets in lattice light-sheet structured illumination microscopy

**DOI:** 10.1117/1.JBO.29.8.086502

**Published:** 2024-07-31

**Authors:** Yu Shi, Tim A. Daugird, Wesley R. Legant

**Affiliations:** a University of North Carolina at Chapel Hill and North Carolina State University, Joint Department of Biomedical Engineering, Chapel Hill, North Carolina, United States; b University of North Carolina at Chapel Hill, Department of Pharmacology, Chapel Hill, North Carolina, United States

**Keywords:** fluorescence microscopy, super-resolution microscopy, lattice light-sheet microscopy, structured illumination microscopy

## Abstract

**Significance:**

Lattice light-sheet structured illumination microscopy (latticeSIM) has proven highly effective in producing three-dimensional images with super resolution rapidly and with minimal photobleaching. However, due to the use of two separate objectives, sample-induced aberrations can result in an offset between the planes of excitation and detection, causing artifacts in the reconstructed images.

**Aim:**

We introduce a posterior approach to detect and correct the axial offset between the excitation and detection focal planes in latticeSIM and provide a method to minimize artifacts in the reconstructed images.

**Approach:**

We utilized the residual phase information within the overlap regions of the laterally shifted structured illumination microscopy information components in frequency space to retrieve the axial offset between the excitation and the detection focal planes in latticeSIM.

**Results:**

We validated our technique through simulations and experiments, encompassing a range of samples from fluorescent beads to subcellular structures of adherent cells. We also show that using transfer functions with the same axial offset as the one present during data acquisition results in reconstructed images with minimal artifacts and salvages otherwise unusable data.

**Conclusion:**

We envision that our method will be a valuable addition to restore image quality in latticeSIM datasets even for those acquired under non-ideal experimental conditions.

## 1 Introduction

Lattice light-sheet microscopy (LLSM) has been widely applied in biological imaging across various scales, spanning from biomolecules to embryos.^1^^–^^3^ This technique offers several advantages over epifluorescence or confocal microscopy, including minimal out-of-focus fluorescence, reduced photobleaching and phototoxicity, and enhanced imaging speed. By utilizing the interference pattern from multiple beams, LLSM improves beam uniformity and axial resolution compared with Gaussian beams.^4^^,^^5^ Typically, to ensure uniform sample illumination and maximize imaging speed, lattice light sheets are laterally dithered to average out modulations due to the interfering beams. However, by stepping the lattice in discrete increments rather than dithering, the same light sheet can be utilized for super-resolution structured illumination microscopy (SIM) yielding improved lateral and axial resolution.^2^ Lattice light-sheet structured illumination microscopy (latticeSIM) also more thoroughly fills out the optical transfer function (OTF) axially compared with dithered lattice illumination, which results in better image quality. However, due to the fixed objective orientation and the lower numerical aperture (NA) of the excitation objective compared with the detection objective, latticeSIM has less resolution improvement than three-dimensional (3D) SIM, and these improvements are restricted to only a single orientation. Nevertheless, the lower photobleaching and phototoxicity of latticeSIM have proven useful for imaging a variety of biological samples.^2^^,^^6^

In addition to the limitations described above, the dual-objective requirement for latticeSIM also introduces the risk of potential misalignment between the excitation pattern and the detection focal plane. Although this is common to all dual-objective light-sheet systems, focus mismatch is particularly detrimental for latticeSIM due to the high NA of excitation and detection and the complexities of the classical SIM reconstruction algorithm,^7^ which can lead to artifacts in the final image.^8^

Although a variety of autofocusing routines^9^^–^^12^ are available to correct the mismatch in axial focus between excitation and detection objectives, these methods face three primary limitations. First, autofocusing often necessitates a separate imaging routine that must be interspersed with a normal timelapse scan. This leads to additional photobleaching and photoxicity as well as slower acquisition rates. Second, sample-induced axial misalignment might vary across the biological sample and even within a single field of view, therefore requiring multiple iterations of autofocusing in different regions. And finally, intensity-based optimizations may not work correctly for the periodic illuminations used in latticeSIM. Hence, there is motivation to develop a posterior approach to detect and correct for misalignments caused by system drift or sample-induced aberrations in latticeSIM. If successful, such an approach could salvage misaligned data, adapt to changes in live samples on the fly, and avoid the need for additional imaging routines.

Inspired by previous publications in opposed objective, interferometric structured illumination (Note 1 in the Supplementary Material),^13^^,^^14^ we propose a method to ascertain the axial mismatch between excitation and detection focal planes in latticeSIM imaging purely from the raw datasets. By measuring the residual phase within the overlap regions among different laterally shifted frequency components, we establish that it is possible to posteriorly determine the axial offset between the illumination pattern and the detection focal plane. We demonstrate the efficacy of this method through simulations and validation using fluorescent beads and biological samples. Once the offset has been determined, we show that reconstructing with transfer functions acquired with the same axial offset as retrieved by our method successfully mitigates artifacts arising from misalignment in the raw data.

## 2 Methods

### 2.1 Theory

For clarity, we adopt here a similar notation to that used by Gustafsson et al.^7^ In fluorescence microscopy, the observed raw data D(r) are the convolution of the sample-emitted fluorescence E(r) and microscope’s detection point spread function (PSF) H(r)

D(r)=(E⊗H)(r). (1)

The emitted fluorescence is the product of the fluorescently labeled sample structure S(r) and the excitation PSF I(r)

E(r)=I(r)S(r). (2)

This can also be written in frequency space as $\overset{˜}{E}(k)=(\overset{˜}{S}⊗\overset{˜}{I})(k)$, where $\overset{˜}{S}(k)$ and $\overset{˜}{I}(k)$ are the Fourier transforms of S(r) and I(r).

**Fig. 1:**
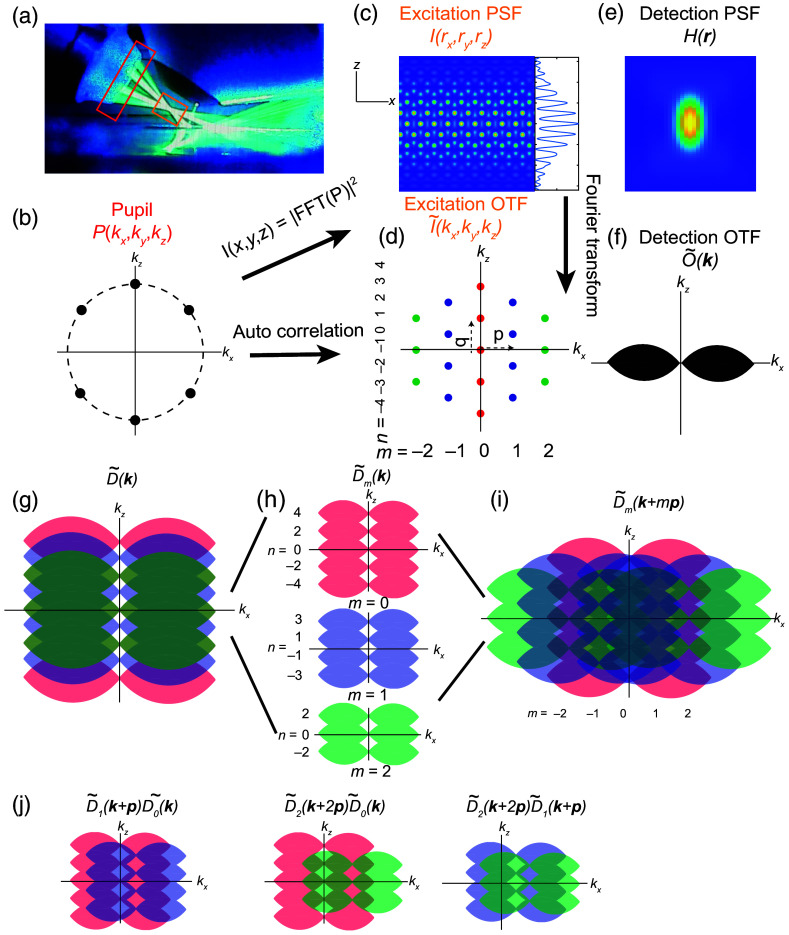
Overview of latticeSIM imaging. (a) Image of the latticeSIM setup. Six beams are emitted by the excitation objective (left) and form a hexagonal interference pattern. The detection objective (right) collects the emitted fluorescence. The red and orange boxes highlight the positions of the pupil and sample planes, respectively. (b) The excitation pattern at the back pupil plane of the excitation objective. (c) The excitation PSF of the hexagonal pattern that illuminates the sample. It is the squared amplitude of the Fourier transform of the pupil function. The inset shows an x-averaged intensity profile along the z axis. (d) The excitation OTF of the hexagonal pattern. It is a Fourier transform of the excitation PSF and an autocorrelation of the pupil function. Different lateral orders (m) are coded by color. Different axial orders (n) are listed as well. (e) Detection PSF H(r). (f) Widefield detection OTF $\tilde{O}(k)$. It is a Fourier transform of the detection PSF. (g)–(i) SIM imaging and reconstruction process. In the SIM images $\tilde{D}(k)$ (g), information components for different lateral orders are shifted to be centered at $k_{x}=0$. In SIM reconstruction, these components are separated (h) and then shifted back to their correct position in frequency space (i). (j) Schematic of the overlap regions between different orders in ${\tilde{D}}_{m}(k)$, left: m=0 and m=1; middle: m=0 and m=2; right: m=1 and m=2.

The latticeSIM excitation pattern I(r) is formed by the interference of multiple beams at the back pupil of the excitation objective. In the case of the hexagonal lattice pattern, the six beams at the back pupil [Figs. 1(a) and 1(b)] will interfere and form an excitation pattern at the sample plane that is the squared magnitude of the Fourier transform of the pupil function [Fig. 1(c)]. Similarly, the excitation OTF $\overset{˜}{I}(k)$ is the Fourier transform of I(r) or the autocorrelation function of the pupil function [Fig. 1(d)]. As is the case for conventional 3D SIM, the excitation pattern can be expressed as a finite sum of “m” components, each of which can be separable into functions that depend solely on the lateral or axial coordinates in real space or frequency space, respectively

$$I(x,y,z)=\underset{m}{\sum }I_{m}(z)J_{m}(x,y)\text{ or }\overset{˜}{I}(k_{x},k_{y},k_{z})=\underset{m}{\sum }{\overset{˜}{I}}_{m}(k_{z})⊗{\overset{˜}{J}}_{m}(k_{x},k_{y}).$$ (3)

For hexagonal lattice illumination, the excitation OTF has a signal at five lateral frequencies and m ranges from −2 to +2, as shown in Fig. 1(d).

Rewriting Eq. (1) with Eq. (2) and the separated components in the excitation PSF (3), it becomes

$$D(r)=(E⊗H)(r)=(S(r)I(r))⊗H(r)=\underset{m}{\sum }\int H(r-r^{\prime })S(r^{\prime })I_{m}(z^{\prime })J_{m}(x^{\prime },y^{\prime })dr^{\prime }.$$ (4)

In the equation above with a spatially invariant PSF, $r^{\prime }$ stands for the reference frame of the specimen, and $r\text{-}r^{\prime }$ stands for the reference frame of the objective lenses. When operating the microscope, the excitation pattern is kept fixed relative to the detection objective focal plane, while the sample is translated along the “z” direction to acquire a 3D volume. Therefore, the axial components of the excitation pattern $I_{m}$ follow the same reference frame as the objectives (same as $H(r-r^{\prime })$) rather than the sample, and $I_{m}(z^{\prime })$ can be replaced by $I_{m}(z-z^{\prime })$. We refer the reader to a full discussion of Gustafsson et al.^7^ for more information about the coordinate reference frames. Equation (4) can then be written as

$$D(r)=\underset{m}{\sum }\int H(r-r^{\prime })I_{m}(z-z^{\prime })S(r^{\prime })J_{m}(x^{\prime },y^{\prime })dr^{\prime }=\underset{m}{\sum }[(HI_{m})⊗(SJ_{m})](r).$$ (5)

And its Fourier transform $\overset{˜}{D}(k)$ is

$$\overset{˜}{D}(k)=\underset{m}{\sum }[\overset{˜}{O}(k)⊗{\overset{˜}{I}}_{m}(k_{z})]\cdot [\overset{˜}{S}(k)⊗{\overset{˜}{J}}_{m}(k_{x},k_{y})],$$ (6)

where $\overset{˜}{O}(k)$ is the Fourier transform of H(r) [Figs. 1(e) and 1(f)], which is the widefield OTF of the detection objective. For SIM illumination, the lateral component $J_{m}(x,y)$ typically follows a simple harmonic where $J_{m}(x,y)=e^{i(2\pi mp\cdot r_{xy}+m\varphi )}$, and ${\overset{˜}{J}}_{m}(k_{x},k_{y})=\delta (k_{xy}-mp)e^{im\varphi }$. Here, p is the fundamental lateral frequency of the illumination pattern, mp are the m’th order lateral harmonics, and $e^{im\varphi }$ defines the lateral phase for J. In lattice SIM, the axial component ${\tilde{I}}_{m}(k_{z})$ also follows a harmonic where $I_{m}(z)=e^{i(2\pi nq\cdot z)}$ and ${\overset{˜}{I}}_{m}(k_{z})=\underset{n}{\sum }\delta (k_{z}-nq)$, where q is the fundamental axial frequency of the illumination pattern and nq is the n’th order axial harmonics. We will further discuss ${\tilde{I}}_{m}(k_{z})$ and its relationship to the microscope alignment later in the paper.

Therefore, Eq. (6) becomes

$$\overset{˜}{D}(k)=\underset{m}{\sum }{\overset{˜}{D}}_{m}(k)=\underset{m}{\sum }\overset{˜}{O}(k)⊗{\overset{˜}{I}}_{m}(k)e^{im\varphi }\overset{˜}{S}(k-mp)=\underset{m}{\sum }{\overset{˜}{O}}_{m}(k)e^{im\varphi }\overset{˜}{S}(k-mp).$$ (7)

Equations (6) and (7) indicate that the resolution is improved by two methods. First, resolution is increased axially through ${\overset{˜}{O}}_{m}(k)=\overset{˜}{O}(k)⊗{\overset{˜}{I}}_{m}(k)$. The overall OTF becomes a series of transfer functions that are the convolution between the widefield detection OTF and the axial part of each excitation OTF order m. And second, sample information components $\overset{˜}{S}(k)$ have been shifted laterally into the detection envelope by the vector mp [Fig. 1(g)]. Note that because the high-resolution axial components are already in their correct locations in frequency space (because the sample is scanned axially as described above), the SIM reconstruction process will need to separate the different “m” lateral components of ${\overset{˜}{D}}_{m}(k)$ [Fig. 1(h)] and then shift the high-frequency components laterally back to their original positions in frequency space [Fig. 1(i)], thus yielding improved resolution (see Gustafsson et al.^7^ for details).

In conventional SIM imaging, the fundamental frequency p and the lateral starting phase φ may be different from when the transfer functions, ${\overset{˜}{O}}_{m}(k)$, are measured and when the sample is imaged. Thus, these parameters are typically fit after data acquisition and during the reconstruction process. This is done by examining the overlap regions among different m components of ${\overset{˜}{D}}_{m}(k)$ in frequency space.

More specifically, once the separated information components of the data have been isolated and shifted to their correct locations in frequency space, the frequency information of the sample $\overset{˜}{S}(k)$ should be identical in the overlapping regions. However, as noted in Eq. (7), the sample information $\overset{˜}{S}(k)$ in the observed data terms ${\overset{˜}{D}}_{m}(k)$ has also been scaled and phase shifted by a corresponding order-specific transfer function ${\overset{˜}{O}}_{m}(k)$ as a result of the physical observation process. Thus, the shifted information components ${\overset{˜}{D}}_{m}(k+mp)$ are not directly comparable to each other. To compensate for this, we multiply the observed data components ${\overset{˜}{D}}_{m}(k+mp)$ by the corresponding transfer function of the other information component in the overlap region. For example, in the overlap regions between m=0 and m=1, we compare

$${\overset{˜}{D}}_{0}(k+0p){{\overset{˜}{O}}^{\prime }}_{1}(k+1p),$$ (8a)

and

$${\overset{˜}{D}}_{1}(k+1p){{\overset{˜}{O}}^{\prime }}_{0}(k+0p),$$ (9a)

as shown in [Fig. 1(j)]. Note that Fig. 1(j) is sketched based on a delta function in real space, or $\overset{˜}{S}(k)$ is a constant; therefore, ${\overset{˜}{D}}_{m}(k)$ and ${\overset{˜}{O}}_{m}(k)$ are equivalent. Here, ${{\overset{˜}{O}}^{\prime }}_{m}(k)$ are the transfer functions obtained when measuring calibration beads at the start of an experiment, e.g., ${{\overset{˜}{O}}^{\prime }}_{m}(k)$ is equivalent to ${{\overset{˜}{D}}^{\prime }}_{m}(k)$ with $\overset{˜}{S}(k)$ replaced by a delta function. Typically for the calibration images, the lateral phase of the excitation pattern, which depends only on the relative position of the bead used for measurement, is set to zero. ${\overset{˜}{D}}_{m}(k)$ is the sample image in frequency space, and because each ${\overset{˜}{D}}_{m}(k)$ already contains a copy of ${\overset{˜}{O}}_{m}(k)$ as described above, Eqs. (8a) and (9a) can each be expanded as

$$\overset{˜}{S}(k){\overset{˜}{O}}_{0}(k+0p){{\overset{˜}{O}}^{\prime }}_{1}(k+1p)e^{i1\varphi },$$ (8b)

and

$$\overset{˜}{S}(k){\overset{˜}{O}}_{1}(k+1p){{\overset{˜}{O}}^{\prime }}_{0}(k+0p)e^{i0\varphi }.$$ (9b)

If we first assume that ${{\overset{˜}{O}}^{\prime }}_{m}(k)={\overset{˜}{O}}_{m}(k)$, which means that the transfer functions when taking the sample image were identical to those obtained when taking the calibration images, then at the correct shift vector p, Eqs. (8) and (9) above will be identical in theory except for a constant phase offset φ. In practice and in the presence of noise, p is determined as the value that maximizes the cross-correlation within the overlapping region. At the correct value for p, the starting phase φ associated with ${\overset{˜}{D}}_{m}(k)$ can be determined by examining the ratio between Eqs. (8) and (9)

$$R_{m_{1},m_{2}(m_{1}\neq m_{2})}=\frac{{\overset{˜}{D}}_{m_{1}}(k+m_{1}p){\overset{˜}{O^{\prime }}}_{m_{2}}(k+m_{2}p)}{{\overset{˜}{D}}_{m_{2}}(k+m_{2}p){\overset{˜}{O^{\prime }}}_{m_{1}}(k+m_{1}p)}=\frac{{\overset{˜}{O}}_{m_{1}}(k+m_{1}p)e^{im_{1}\varphi }\overset{˜}{S}(k){\overset{˜}{O^{\prime }}}_{m_{2}}(k+m_{2}p)}{{\overset{˜}{O}}_{m_{2}}(k+m_{2}p)e^{im_{2}\varphi }\overset{˜}{S}(k){\overset{˜}{O^{\prime }}}_{m_{1}}(k+m_{1}p)}=e^{i(m_{1}-m_{2})\varphi }.$$ (10)

Note that this ratio is independent of sample information and is constant throughout the entire overlap region. Therefore, φ can be extracted using complex linear regression upon the two terms, and the slope carries the information of the starting phase φ.

In contrast to conventional single-objective SIM, in latticeSIM the axial components in ${\overset{˜}{I}}_{m}(k)$ may also acquire a phase term that is dependent on δz—the physical offset between the detection and excitation reference planes. In this case, assuming that the illumination pattern along z also follows a harmonic distribution, the axial components of the excitation pattern can be defined as:

$${\overset{˜}{I}}_{m}(k_{z})=\underset{n}{\sum }\delta (k_{z}-nq)e^{i2\pi (\delta z*nq)}.$$ (11)

Here, q is the fundamental frequency along the axial direction, and n is a function of m. Specifically, as shown in Fig. 1(d)

$$n=\{\begin{array}{ll} 0,\pm 2 & \text{when }m=\pm 2\\ \pm 1,\pm 3 & \text{when }m=\pm 1\\ 0,\pm 2,\pm 4 & \text{when }m=0 \end{array},$$

**Fig. 2:**
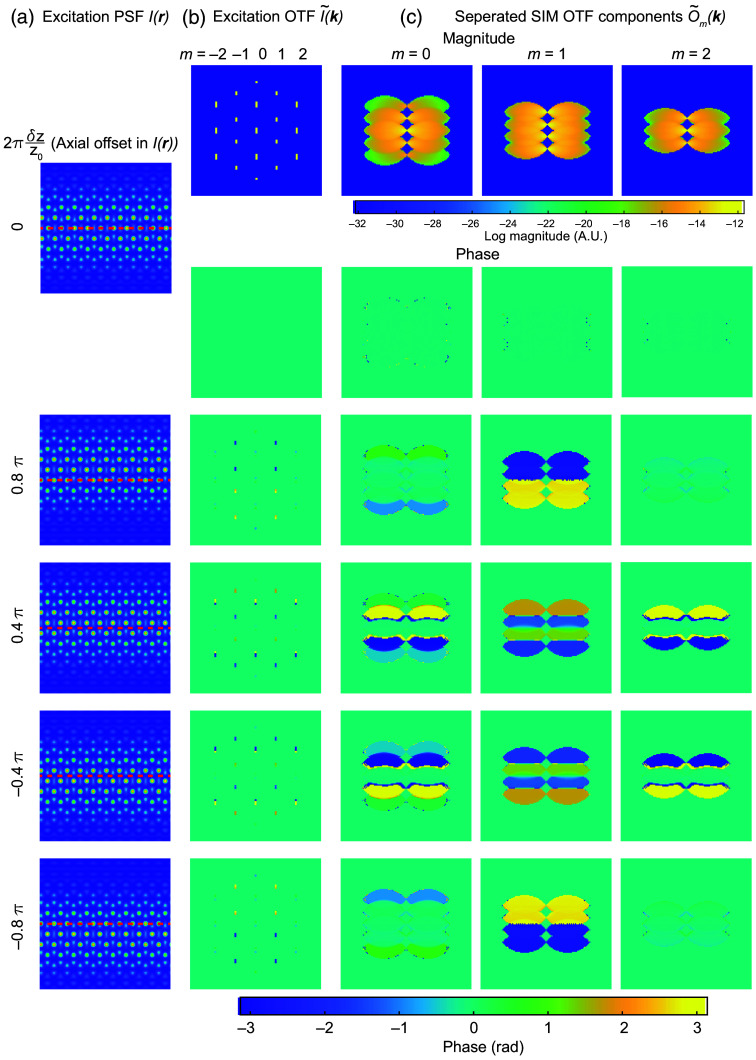
Excitation and SIM OTFs with different axial offsets. (a) Excitation PSF I(r) in real space. The number at the left indicates the offset from the simulated detection objective focal plane. (b) Excitation OTF $\overset{˜}{I}(k)$ in frequency space. The first row shows the magnitude. The rows underneath show the phase under different axial offsets. (c) Separated SIM transfer functions ${\overset{˜}{O}}_{m}(k)$ [same as Fig. 1(h)] showing the magnitude and phase under different excitation pattern axial offsets.

2π(δz*nq) is the phase shift in the excitation OTF that is caused by the physical misalignment δz between the excitation pattern and the detection objective focal plane [Figs. 2(a) and 2(b)]. Because the overall OTF is the convolution of the detection OTF with the excitation OTF [Fig. 1(g)], any phase term in the excitation OTF manifests in the overall transfer functions of the instrument [Fig. 2(c)]. Therefore, the amount of axial misalignment of the excitation pattern can, in theory, be extracted if we could determine the relative contribution to the phase information in ${\overset{˜}{D}}_{m}(k)$ that was due to the transfer functions ${\overset{˜}{O}}_{m}(k)$ when the image was acquired.

**Fig. 3:**
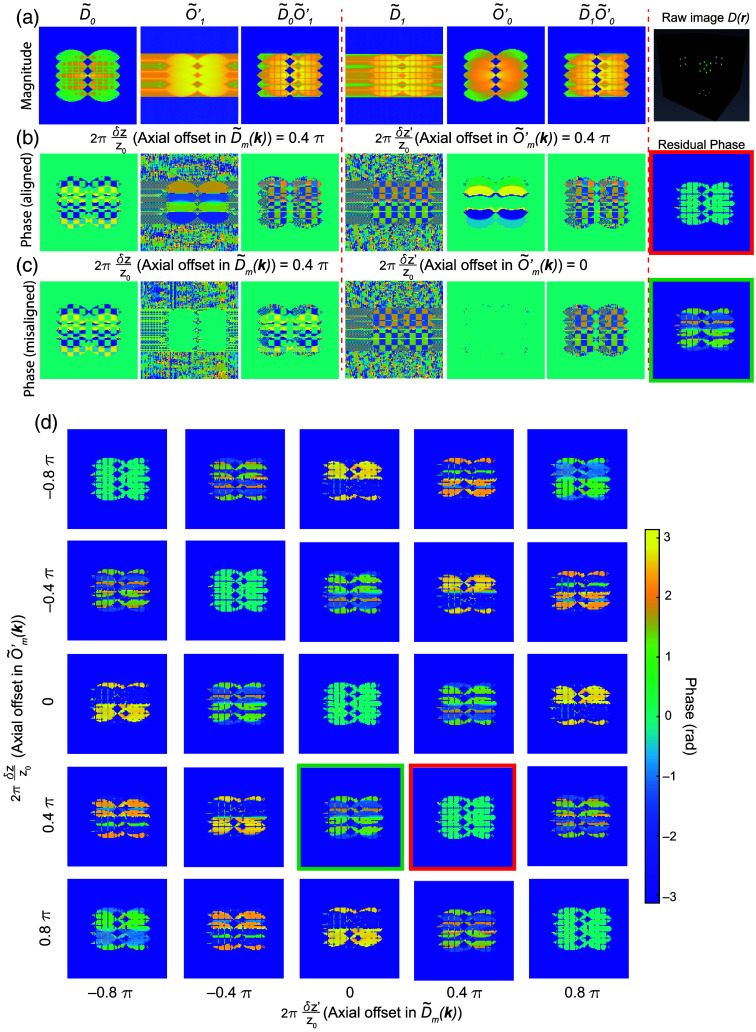
Residual phase in OTF overlaps reflects the axial offset. (a) Columns 1 to 3: Magnitude of the zeroth information component (${\tilde{D}}_{0}$), the first SIM transfer function (${{\overset{˜}{O}}^{\prime }}_{1}$) after being shifted to the correct position in frequency space, and the magnitude of the product of the two. Here, the SIM image is composed of eight beads. Columns 4 to 6: Magnitude of the first information component in the SIM image after being shifted to the correct position in frequency space (${\tilde{D}}_{1}$), the zeroth SIM transfer function (${{\overset{˜}{O}}^{\prime }}_{0}$), and the magnitude of the product of the two. (b) The phase of the corresponding columns in panel (a). In this case, both the SIM image ${\overset{˜}{D}}_{m}(k)$ and transfer functions (${{\overset{˜}{O}}^{\prime }}_{m}(k)$) have been simulated with a 0.4 π axial offset between the excitation and detections. The rightmost column shows the residual phase in the overlapped region as measured via the ratio in Eq. (12). Panel (c) is the same as panel (b) but in this case, the transfer function ${{\overset{˜}{O}}^{\prime }}_{m}(k)$, does not have the corresponding 0.4 π axial offset as ${\overset{˜}{D}}_{m}(k)$. (d) Residual phase as computed via Eq. (12) for different axial offsets between the excitation and detection planes in simulated SIM images (different columns) and simulated transfer functions (different rows).

However, the transfer functions ${\overset{˜}{O}}_{m}(k)$ are not typically accessible in raw images, and as with the lateral starting phase above, the axial offset may vary from when the transfer functions are calibrated at the beginning of an experiment and when a given image is acquired. For example, as shown in Eq. (7), the phase of the raw images ${\overset{˜}{D}}_{m}(k)$ composed of eight beads [rightmost column in Fig. 3(a)] is a mixture of illumination and sample information [Figs. 3(a) and 3(b)]. Therefore, to retrieve the axial misalignment, we need to first cancel out the sample information $\overset{˜}{S}(k)$ from the observed image $\overset{˜}{D}(k)$. This can be achieved by the same approach described in Eq. (10). If we include an axial offset which leads to phase ramp in ${\tilde{I}}_{m}(k_{z})$ as described in Eq. (11), and assume that there may be a different axial offset δz and $\delta z^{\prime }$ when imaging the sample or calibration beads respectively, then Eq. (10) then becomes

$$R_{m_{1},m_{2}(m_{1}\neq m_{2})}=\frac{\overset{˜}{O}(k+m_{1}p)⊗{\overset{˜}{I}}_{m_{1}}(k_{z})e^{im_{1}\varphi }\overset{˜}{S}(k)\overset{˜}{O}(k+m_{2}p)⊗{\overset{˜}{I^{\prime }}}_{m_{2}}(k_{z})}{\overset{˜}{O}(k+m_{2}p)⊗{\overset{˜}{I}}_{m_{2}}(k_{z})e^{im_{2}\varphi }\overset{˜}{S}(k)\overset{˜}{O}(k+m_{2}p)⊗{{\overset{˜}{I}}^{\prime }}_{m_{1}}(k_{z})}\;=\frac{\underset{n_{1}}{\sum }\overset{˜}{O}(k+m_{1}p)⊗\delta (k_{z}-n_{1}q)e^{i(2\pi \delta z*n_{1}q+m_{1}\varphi )}\underset{n_{2}}{\sum }\overset{˜}{S}(k)\overset{˜}{O}(k+m_{2}p)⊗\delta (k_{z}-n_{2}q)e^{i(2\pi \delta z^{\prime }*n_{2}q+m_{2}\varphi )}}{\underset{n_{2}}{\sum }\overset{˜}{O}(k+m_{2}p)⊗\delta (k_{z}-n_{2}q)e^{i(2\pi \delta z*n_{2}q+m_{2}\varphi )}\underset{n_{1}}{\sum }\overset{˜}{S}(k)\overset{˜}{O}(k+m_{1}p)⊗\delta (k_{z}-n_{1}q)e^{i(2\pi \delta z^{\prime }*n_{1}q+m_{1}\varphi )}}\;=\frac{\overset{˜}{S}(k)\sum_{n_{1}}\overset{˜}{O}(k+m_{1}p+n_{1}q)e^{i(2\pi \delta z*n_{1}q+m_{1}\varphi )}\sum_{n_{2}}\overset{˜}{O}(k+m_{2}p+n_{2}q)e^{i(2\pi \delta z^{\prime }*n_{2}q+m_{2}\varphi )}\;}{\overset{˜}{S}(k)\sum_{n_{2}}\overset{˜}{O}(k+m_{2}p+n_{2}q)e^{i(2\pi \delta z*n_{2}q+m_{2}\varphi )}\sum_{n_{1}}\overset{˜}{O}(k+m_{1}p+n_{1}q)e^{i(2\pi \delta z^{\prime }*n_{1}q+m_{1}\varphi )}}.\;$$ (12)

Note that here, we have expanded ${\overset{˜}{O}}_{m}(k)=\overset{˜}{O}(k)⊗{\overset{˜}{I}}_{m}(k_{z})$. We also use ${\tilde{I}}_{m}(k_{z})$ and ${{\overset{˜}{I}}^{\prime }}_{m}(k_{z})$ to denote the axial component of illumination when imaging the sample and the calibration beads, respectively. Here, we assume there is no phase contributed from the widefield detection OTF, as it can be corrected by proper alignment or adaptive optics. Note that $R_{m_{1},m_{2}(m_{1}\neq m_{2})}$ is valid only in regions where ${\overset{˜}{D}}_{m_{1}}(k+m_{1}p){{\overset{˜}{O}}^{\prime }}_{m_{2}}(k+m_{2}p)$ and ${\overset{˜}{D}}_{m_{2}}(k+m_{2}p){{\overset{˜}{O}}^{\prime }}_{m_{1}}(k+m_{1}p)$ are non-zero, which are indicated by the magnitude of the products shown in Fig. 3(a) ($m_{1}=0$ and $m_{2}=1$). For a specific combination of $\left(m_{1},n_{1}\right)$ and $\left(m_{2},n_{2}\right)$, the residual phase, which is the phase of $R_{m_{1},m_{2}(m_{1}\neq m_{2})}$, can be expressed as

$$\Phi_{m_{1},n_{1},m_{2},n_{2}(m_{1}\neq m_{2})}=2\pi q(n_{1}-n_{2})(\delta z-\delta z^{\prime })+(m_{1}-m_{2})\varphi .$$ (13)

**Fig. 4:**
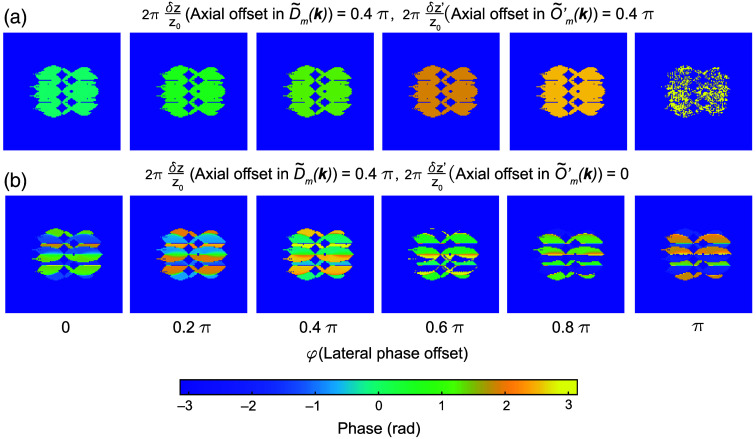
Mixing between axial and lateral phase terms in the overlap regions. (a) Residual phase computed via Eq. (12) for a single bead image when both the SIM image ${\overset{˜}{D}}_{m}(k)$ and simulated transfer functions ${{\overset{˜}{O}}^{\prime }}_{m}(k)$, have a 0.4 π axial offset between the excitation and detection objectives. Different columns show the effects of different lateral offsets in the excitation pattern $\overset{˜}{I}(k)$ used when simulating the data ${\overset{˜}{D}}_{m}(k)$ and transfer functions ${{\overset{˜}{O}}^{\prime }}_{m}(k)$. Panel (b) is the same as panel (a) but when utilizing a simulated transfer function with no axial offset.

**Fig. 5:**
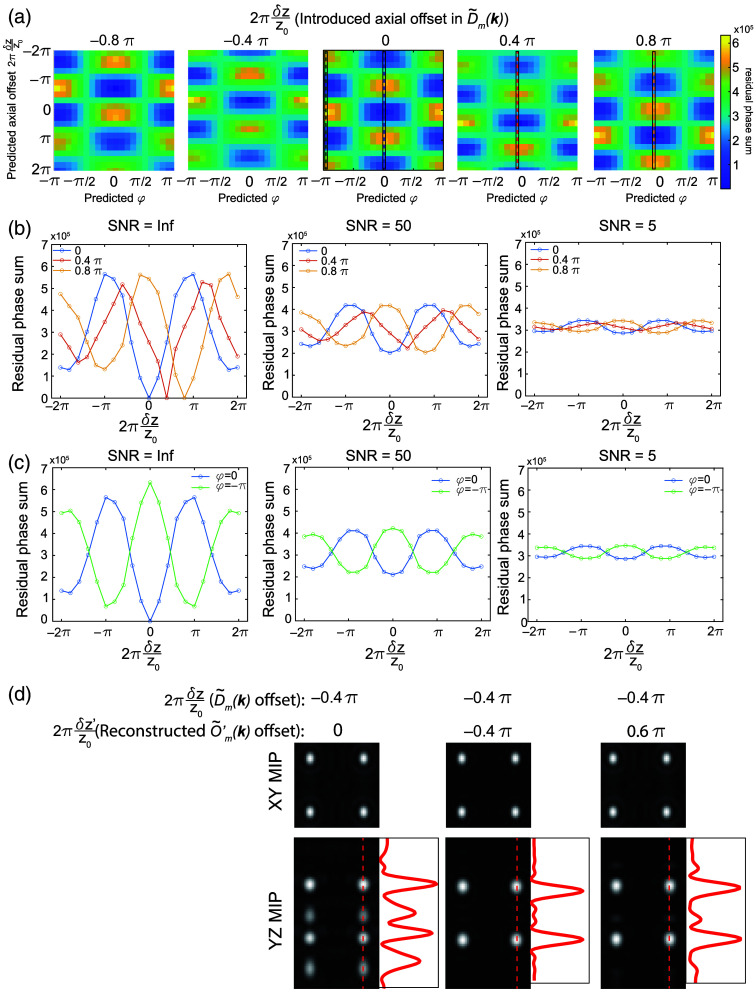
Retrieving the axial offset between excitation and detection planes in simulated data. (a) 2D maps of the summed residual phase in the overlap regions with different simulated axial (vertical axis) and lateral (horizontal axis) offsets in the transfer functions ${{\overset{˜}{O}}^{\prime }}_{m}(k)$ used for reconstruction. The simulated applied axial offset in the SIM image ${\overset{˜}{D}}_{m}(k)$ is shown on the top of each image. (b) Line profiles in panel (a) under different SNRs assuming that the lateral offset between ${\overset{˜}{D}}_{m}(k)$ and ${{\overset{˜}{O}}^{\prime }}_{m}(k)$ is zero. Different colors indicate different applied axial offsets in the SIM image as indicated by the dashed lines in panel (a). Blue: no axial offset, red: 0.4 π axial offset, orange: 0.8 π axial offset. (c) Line profiles in panel (a) under different SNR when the lateral offset between ${\overset{˜}{D}}_{m}(k)$ and ${{\overset{˜}{O}}^{\prime }}_{m}(k)$ is either zero (blue) or −π (green). This illustrates the residual phase differences between the optimal pattern and the local minimum that is one-half period off both axially and laterally. (d) Reconstructed SIM image of four beads that were simulated with a −0.4 π axial offset between the excitation illumination and the detection focal plane. The different columns show the effects of reconstructing with transfer functions that assume no axial offset (left), the correctly identified axial offset (middle), and an axial offset that is one-half period off laterally and axially (right). The red curves show the intensity line profile along the red dashed lines.

Equation (13) is the sum of two terms. The first term is determined by the axial offset between the excitation and detection foci, will be zero if $\delta z=\delta z^{\prime }$ [Figs. 3(b) and 3(c)], and is a function of $k_{z}$ (because it contains the $k_{z}$-oriented vector). The second term is due to the lateral phase φ in the illumination pattern and is a constant across the overlap region [the same as Eq. (10)]. This has two important implications for the reconstruction process. The first is that it creates a need to explicitly account for the axial phase offset during reconstruction. For light-sheet illumination, the delta functions of the hexagonal lattice in the pupil are extended into lines or Gaussian profiles along the $k_{z}$ direction, corresponding to the axial confinement of the light sheet at the sample. This complicates the simplified version of the residual phase shown in Eq. (13) and makes it challenging to derive an analytical solution. In practice, to extract the axial offset δz from the sample images, we can collect a gallery of transfer functions with different known axial offsets $\delta z^{\prime }$ and then identify for which measured offset $\Phi_{m_{1},m_{2}}$ reaches a minimum value at $\delta z^{\prime }=\delta z$ [Fig. 3(d)]. The corresponding transfer functions can then be used to reconstruct the SIM images and will yield minimized artifacts due to axial misalignment between the excitation and detection focus. The second implication of Eq. (13) is that it shows that the conventional method for estimating the lateral phase offset φ as described in Eq. (10) via complex linear regression will fail as the residual phase $\Phi_{m_{1},m_{2}}$ is no longer constant in the overlap region as assumed by conventional SIM reconstruction. As illustrated in Fig. 4, the residual phase Φ is constant only when the first term in Φ is zero ($\delta z=\delta z^{\prime }$, [Fig. 4(a)]. Whenever $\delta z\neq \delta z^{\prime }$, Φ will depend on $q(n_{1}-n_{2})$. This means that the ratio $R_{m_{1},m_{2}}$ is no longer a constant and ${\overset{˜}{D}}_{m_{1}}(k+m_{1}p){{\overset{˜}{O}}^{\prime }}_{m_{2}}(k+m_{2}p)$ is no longer linearly related to ${\overset{˜}{D}}_{m_{2}}(k+m_{2}p){{\overset{˜}{O}}^{\prime }}_{m_{1}}(k+m_{1}p)$. Therefore, complex linear regression applied in conventional SIM reconstruction will fail. In Secs. 3.1–3.4, we address both of these aspects, by generating a two-dimensional (2D) map of the residual phase $\Phi_{m_{1},m_{2}}$ that is composed of δz and φ and use the minimum to simultaneously extract the axial and the lateral offset needed for proper reconstruction [Fig. 5(a)]. This method can also be extended to another illumination lattice (e.g., square lattice) used in lattice SIM (Fig. S1 in the Supplementary Material).

### 2.2 Simulation and Image Analysis

#### 2.2.1 Simulation of structural illumination microscopy images

All simulated datasets were constructed in Matlab (The Mathworks, Natick, Massachusetts, United States) using the previously published code base.^4^ To adapt this code to generate SIM images, we generated the detection PSF using the Debye approximation. Specifically, we first simulated the back pupil of a cone in a sphere with NA = 1.0 and a refractive index of 1.33 and then Fourier transformed it into real space. We simulated the excitation PSF following a similar approach but using a pupil of six evenly spaced vertical lines within an annular ring [Fig. 1(b), NA = 0.55/0.5]. We simulated five copies of excitation PSFs whose lateral starting phase offset φ evenly covers the entire period by introducing a phase gradient along $k_{x}$ in the pupil function. Because the physics of image formation are continuous, we up sampled by a factor of 2 when simulating the forward imaging process. The excitation OTF of the hexagonal pattern was dissected into different lateral orders as in Eq. (3). We then followed Eqs. (5) and (6) to simulate the raw SIM images. Briefly speaking, the axial components of the excitation PSF $I_{m}(r_{z})$ were multiplied with the detection PSF H(r), and the lateral components of the excitation OTF $J_{m}(r_{x},r_{y})$ were multiplied with the Fourier transform of sample information S(r). The Fourier transform of $HI_{m}(r)$ and $J_{m}S(r)$ was then multiplied in frequency space and Fourier transformed back to real space to generate raw images $D_{m}(r)$. Different orders of $D_{m}(r)$ were then summed to form the final raw image D(r), which was downsampled to replicate image discretization onto camera pixels. To simulate different axial offsets of the excitation pattern, we applied a phase gradient along $k_{z}$, respectively, in the pupil function when we simulated the excitation PSF. Further details and a step-by-step walkthrough of the simulation process are provided in a Matlab Live script included in the accompanying GitHub repository.

#### 2.2.2 Simulation of randomly distributed beads

We simulated 3D volumes of randomly distributed bead images using the approach described by Shi et al.^4^ In brief, we first simulated a 3D volume with points randomly distributed as S(r) in Eq. (6). We then follow the pipeline above to simulate the corresponding SIM images. To simulate images of different signal-to-noise ratios (SNRs), we added a Gaussian noise floor in the simulated images.

#### 2.2.3 SIM reconstruction

SIM reconstruction was performed using code from cudasirecon,^15^ following the algorithm described by Gustafsson et al.^7^ and using the parameters provided in the settings file in the accompanying GitHub repository.

### 2.3 Experiments

#### 2.3.1 Microscopy setup

The optical path for latticeSIM is based on a modified version of the instrument described by Chen et al.^2^ Key modifications relevant to this work are the use of a 0.6 NA excitation lens 404 (Thorlabs, TL20X-MPL, Newton, New Jersey, United States), and 1.0 NA detection lens (Zeiss, Objective W “Plan-Apochromat” x20/1.0, model # 421452-9800, Oberkochen, Germany).

LatticeSIM was operated with a hexagonal lattice pattern with a maximum and minimum NA of 0.55 and 0.5 at the back pupil, respectively. This pattern yields a period of 1.202  μm in x and 2.13  μm in z. For each z plane, we collected five copies of images, each with illumination pattern shift 0.24  μm in x and covering the entire 1.202  μm period.

#### 2.3.2 Immunofluorescence

We cultured retinal pigment epithelium (RPE) cells (RRID: CVCL_4388, ATCC) in Dulbecco’s Modified Eagle Medium (Gibco 11965-092, Grand Island, New York, United States) with 10% FBS (VWR: 1500-050) and 1% (v/v) 10,000 U/ml Penicillin-Streptomycin (Gibco 15140-122). For fixed cell imaging, we incubated RPE cells with either 250 nM mitotracker orange (Thermo Fisher, M7510, Waltham, Massachusetts, United States) in culture media for mitochondria staining or a 1000× dilution manufacturer recommended stock concentration of SPY555-DNA (Cytoskeleton Inc., Denver, Colorado, United States) in culture media for histone staining for 30 min. We then fixed cells with 4% Paraformaldehyde (Electron Microscopy Sciences, 15710, Hatfield, Pennsylvania, United States) and 8 nM/ml sucrose (Sigma, S7903, Garner, North Carolina, United States) in cytoskeleton buffer (composed of 10 mM MES, 138 mM KCl, 3 mM MgCl, and 2 mM EGTA) for 20 min at room temperature.

#### 2.3.3 Hydrogel preparation

Polyacrylamide substrates containing fluorescent beads were prepared as described by Tse and Engler.^16^ Briefly, a mix of 40% acrylamide and 2% bis-acrylamide was prepared in 10 mL diH20 to an estimated stiffness of 8 kPA. 1/1000 by volume of carboxylate red fluorosphere fluorescent beads (580 nm/605 nm excitation/emission wavelength, Thermo Fisher, F8801) were added to 495  μL of the mix and placed in a vacuum desiccator for 15 min to reduce dissolved oxygen before addition of 5  μL of 10% by weight of APS (freshly prepared in ${\mathrm{diH}}_{2}O$) and 0.5  μL of TEMED. Immediately, 30  μL of the solution was carefully pipetted onto a 25 mm glass coverslip that was previously activated with 97% APTES and 0.5% Glutaraldehyde, and then, a 12 mm glass coverslip was placed on top of the solution. After polymerization, the coverslip sandwich was immersed in ${\mathrm{diH}}_{2}O$ water for 5 min before a razor blade was used to carefully peel the 12 mm glass coverslip off of the 25 mm coverslip. The PAA substrate on a 25 mm coverslip was then immersed in diH20 and stored in a 4°C fridge until use.

#### 2.3.4 Measurements of light-sheet offset in hydrogel

To measure the extent to which a light sheet is deflected when imaging through thick hydrogels, we embedded 100 nm diameter red fluorescent beads (580  nm/605  nm excitation/emission wavelength, Thermo Fisher, F8801) inside the hydrogel. To measure the light sheet offset at different depths in the sample, we followed a similar approach as described by Shi et al.^4^ Briefly speaking, we first axially scanned the excitation profile relative to the detection focal plane and plotted the integrated fluorescence signal from a small (3 pixel) region around each bead in the field of view. The peak of this plot defines the center of the excitation profile relative to the position of each bead underneath the sample. We then determined the position of each bead relative to the detection objective focal plane by scanning the coverslip together with the light-sheet illumination along the optical axis of the detection objective (equivalent to widefield illumination). The offset of the excitation pattern relative to the detection objective focal plane is then computed from these plots by comparing the positions of the light sheet relative to the bead and the position of the bead position relative to the focal plane.

## 3 Results

### 3.1 Minimizing Residual Phase Yields the Transfer Functions with the Correct Axial Offset in Simulation

To test whether our methodology can reliably extract the axial misalignment of the excitation pattern from simulated images, we first simulated a transfer function library ${{\overset{˜}{O}}^{\prime }}_{m}(k)$ and bead images ($\overset{˜}{D}(k)$) with different excitation pattern axial offsets. We quantify the sum of the residual phase in the overlap region between different orders in $\Phi_{01}$ and $\Phi_{12}$ as the metric to minimize. As shown in Eq. (12), the phase of $\Phi_{01}$ and $\Phi_{12}$ will be a function of axial offset $\delta z^{\prime }$ and the lateral offset φ. To determine both the axial offset and lateral phase simultaneously, we search for the minimum in $\Phi_{01}$ and $\Phi_{12}$ in a 2D residual phase map corresponding to each pairing of axial and lateral offset [Fig. 5(a)]. As illustrated in Fig. 5(b), the axial offset corresponding to the minimum value in the 2D residual phase map matches the misalignment that we introduced in the simulation. Moreover, we noticed that as the SNR decreases, the modulation depth of the residual phase array also decreases, making it harder to distinguish the true light-sheet axial focus from the position that is one full period off [Fig. 5(b)].

Another caveat of this methodology is that the aligned [blue line in Fig. 5(c)] and pi offset along axial and lateral direction [green line in Fig. 5(c)] are both local minima in the 2D phase map. The difference between the two minima also decreases as the SNR decreases [Fig. 5(c), blue and green curves]. This means that with increasing noise, our method will have trouble distinguishing between transfer functions that are laterally and axially offset by half of a period rather than honing in on the correct axial offset. We next tested how this will affect the performance of SIM reconstruction. We simulated an image of eight beads whose excitation axial focal plane is 0.4 π below the detection focal plane, and we reconstructed this simulated image with the transfer functions that generated the minimum variance in residual phase (−0.4 π offset) or the transfer functions that are a half a period off axially (0.6 π offset). As shown in Fig. 5(d), both reconstructed images show minimum artifacts compared with the image reconstructed with calibration transfer functions that did not account for axial misalignment. For axially periodic illumination patterns, there will be no difference between the two minima as they represent different nodes in the lattice. However, the Gaussian envelope associated with the light-sheet confinement differentiates these two local minima and may cause noticeable artifacts for tightly confined beams. Therefore, in practice to minimize this effect in the experimental data, we limited the lateral offset search range to a minimum between −π/2 and π/2.

### 3.2 Validation with Fluorescent Beads

**Fig. 6:**
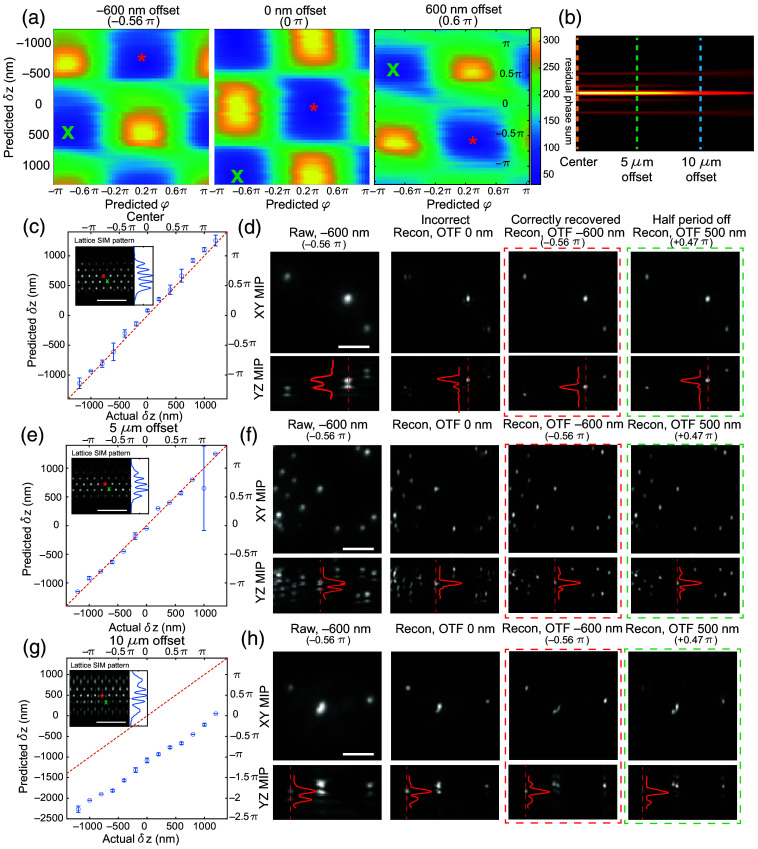
Retrieving the axial offset between excitation and detection planes in experimental data with point-like objects. (a) 2D maps of the summed residual phase in the overlap regions with different experimentally obtained axial (vertical axis) and lateral (horizontal axis) offsets in the transfer functions ${{\overset{˜}{O}}^{\prime }}_{m}(k)$ used for reconstruction. The applied axial offset in the experimentally obtained SIM data is shown at the top of each image. The red (*) shows the minimum residual phase measured between −0.5 π and 0.5 π lateral offsets. The green (x) shows the local minima that are located one-half period off axially and laterally. (b) Different offsets in the excitation pattern along the beam propagation direction. Orange: no offset, green: 5  μm offset, blue: 10  μm offset. (c) Comparison between the applied (horizontal axis) and predicted (vertical axis) axial offset between the excitation and detection focal planes in SIM image taken with the sample located at the center of the beam [orange line in panel (b)]. The inset shows the illumination pattern I(r). Red (*) and green (x) correspond to the two minima in panel (a). Scale bar=5  μm. (d) Raw and reconstructed SIM images of fluorescent beads taken under the same settings as in panel (c) and with a −600  nm axial offset between the excitation and detection focal planes. The first column shows the raw image, the second column shows the image reconstructed with transfer functions using the correctly predicted axial offset (−600  nm), the third column shows the image reconstructed with transfer functions whose excitation and detection focal planes are aligned, the fourth column shows the image reconstructed with transfer functions that utilize an axial offset that is one-half a period off from the predicted value (i.e., 500 versus −600  nm). Scale bar=2  μm. Panels (e) and (f) are the same as panels (c) and (d) but images are taken with 5  μm offset from the beam center along the propagation direction. Panels (g) and (h) are the same as panels (c) and (d), but images are taken with 10  μm offset from the beam center along the propagation direction. The red curves show the intensity line profile along the red dashed lines.

After testing the approach via simulations, we next experimentally validated it with fluorescent beads. We deliberately introduced a known axial offset between the lattice light sheet and the detection objective focal plane and tested whether the minimum position in the 2D phase map could recover the applied offset. As illustrated in Fig. 6(a), the minimum position in the 2D phase map within the −π/2 to π/2 lateral phase offset range [indicated by the red asterisks in Fig. 6(a)] matches the introduced axial offset, thus demonstrating that our simulated results also transfer over to experimental datasets. In latticeSIM, the shape of the illuminated lattice will vary as the beam diverges along the propagation direction of the beam. Therefore, after validating that the method works when the sample is centered along the propagation direction of the excitation pattern, we next tested the performance when the sample is displaced along this axis [Figs. 6(b)–6(h)]. We applied a known axial offset upon the illumination light sheet (the value of the x-axis in Figs. 6(c), 6(e) and 6(g). We then extracted the axial position of the minimum in the 2D phase map and compared the predicted axial offsets (blue circles in Figs. 6(c), 6(e), and 6(g)] to the ideal fit of the experimental offsets (red dashed lines). We found that at the propagation focal position [Fig. 6(c), orange line in Fig. 5(b)] or 5  μm off from the propagation focal position [Figs. 6(e), green line in Fig. 5(b)], we can successfully retrieve the axial offset within the range of 1.2  μm (1.1 π) above or below the detection focal plane (one full lattice period along the axial direction is 2.13  μm), as the blue circles matched well with the red dashed lines. We investigated the performance of SIM reconstruction using the predicted transfer function from our methods. As shown in Figs. 6(d) and 6(f), the raw lattice light-sheet images with a 600 nm (0.56 π) axial offset between the focal plane of the excitation and the detection objectives have clear ghost copies [YZ max intensity projection (MIP) in the first column of Figs. 6(d) and 6(f)], and similar effects were observed in the SIM images that were reconstructed with “zero-offset” transfer functions [the second column of Figs. 6(d) and 6(f)]. The reconstructed images using transfer functions that were generated with the computationally predicted excitation offset (y values of blue circles in Figs. 6(c) and 6(e), reconstructed images shown in the third column of Figs. 6(d) and 6(f)] yield a less aberrated image. Moreover, images reconstructed with transfer functions that are half a period off axially and laterally from the predicted positions [green crosses in Figs. 6(a) and 6(c) inset] yield comparable quality [Figs. 6(d) and 6(f), fourth column].

However, we also noticed that when imaging with an excitation pattern that is 10  μm away from the focus along the beam propagation direction [Figs. 6(g) and 6(h), the blue line in Fig. 6(b)], the predicted axial offset locked in on one that is a half period off laterally and axially [Fig. 6(g)]. We suspect that this is because the aberrations in the excitation pattern for this condition actually represent a combination of a linear phase ramp due to the axial beam offset and a defocus phase term due to the propagation offset. A failure to account for the defocus phase term likely reduced the ability to distinguish between the two local minima that are one-half period away from each other. Furthermore, as in Fig. 6(h), images reconstructed with transfer functions having the correct axial offset [third column in Fig. 6(h)] or half a period off [fourth column in Fig. 6(h)] both yield symmetric side lobes in the YZ MIP. However, we also noticed that, even at the corrected axial focus, these reconstructed images were more aberrated compared with the ones in Figs. 6(d) and 6(f) where the sample was at the propagation focus of the light sheet. We believe that this is likely because the structured illumination pattern is more distorted compared with the ones at the focal position or 5  μm off from the focal position. Therefore the weighting among different frequency components of ${\overset{˜}{O}}_{m}(k)$ is different from ${{\overset{˜}{O}}^{\prime }}_{m}(k)$, which will yield artifacts during the Wiener deconvolution step in image reconstruction. However, the fact that the side lobes are symmetric in Fig. 6(h) still indicated that our method can still successfully predict the amount of axial offset given the raw image.

### 3.3 Validation with Adherent Cells

**Fig. 7:**
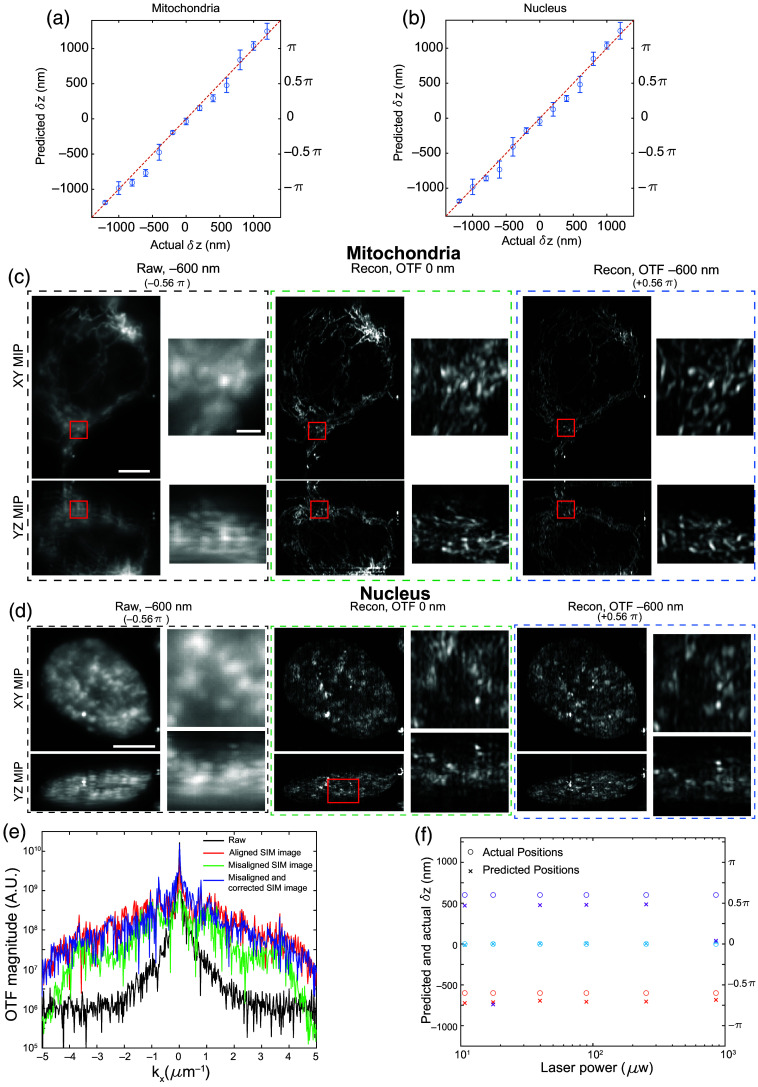
Demonstration of the method with adherent cells. (a), (b) Comparisons between the experimentally applied (horizontal axis) and computationally predicted (vertical axis) axial offset between the excitation and detection focal plane when imaging cellular mitochondria (a) and the cell nucleus (b). (c) Raw and reconstructed SIM images of mitochondria taken with the cell aligned at beam propagation focus [orange line in Fig. 6(b)] and with a −600  nm axial offset between the excitation and detection focal planes. The first column shows the effective dithered lattice image by summing the images from each of the five lateral phase steps, the second column shows the image reconstructed with transfer functions whose excitation and detection focal planes are aligned, and the third column shows the image reconstructed with transfer functions that used a computationally identified axial offset of −600  nm. Scale bar=5  μm. Insets show a zoomed-in view of the red box, inset scale bar=2  μm. Panel (d) is the same as panel (c) but with images of the cell nucleus. (e) Linecuts of the power spectrums for the five-phase summed raw images (black), and images that were acquired and reconstructed under the following settings: ${\overset{˜}{D}}_{m}(k)=0\;\mathrm{nm}$ and ${{\overset{˜}{O}}^{\prime }}_{m}(k)=0\;\mathrm{nm}$ (red); ${\overset{˜}{D}}_{m}(k)=-600\;\mathrm{nm}$ and ${{\overset{˜}{O}}^{\prime }}_{m}(k)=0\;\mathrm{nm}$ (green); ${\overset{˜}{D}}_{m}(k)=-600\;\mathrm{nm}$ and ${{\overset{˜}{O}}^{\prime }}_{m}(k)=-600\;\mathrm{nm}$ (blue). (f) Comparisons between the applied (circles) and predicted (crosses) axial offsets between the excitation and detection focal plane in images of mitochondria taken with different laser power at the back pupil of the excitation objective. The test was run at three different experimentally applied offsets: −600  nm (orange), 0 nm (cyan), and +600  nm (purple).

To benchmark the performance of our method on biological samples, we imaged two subcellular structures in RPE cells: mitochondria and nuclei. As illustrated in Figs. 7(a) and 7(b), our method can successfully retrieve the introduced axial excitation offset in these two structures over the range of −1.2  μm to +1.2  μm (−1.1 π to +1.1 π). Similar to our benchmark with beads, raw lattice light-sheet images taken with a 600 nm offset between the excitation focal plane and the detection focal plane show ghost copies in the YZ MIP, and the same ghost copies appeared in the reconstructed images that were processed with “zero-offset” transfer functions [Figs 7(c) and 7(d), first and second columns]. Moreover, using transfer functions with the correctly predicted axial offset again yielded an image with minimal aberration [Figs. 7(c) and 7(d), third column]. The same trends can be observed in linecuts of the image power spectrums where the SIM image reconstructed with the corresponding transfer functions [with or without axial offset between the excitation and detection focal planes, red and blue curves in Fig. 7(e)] have better high-frequency support compared with SIM images that were reconstructed with incorrect transfer functions. Overall, these results highlight that our method can successfully retrieve the axial offset from the raw latticeSIM images and yield reconstructions with minimal aberrations even when imaging complex 3D structures in cells.

Another factor that will affect the performance of our method is SNR. Because SNR may be low when imaging biological samples where phototoxicity and photobleaching need to be minimized, we tested our methods with cellular mitochondria using different laser powers. As shown in Fig. 7(f), the accuracy of our approach did not degrade substantially as the applied laser power decreased. This indicated that our method is robust across SNR, at least over the range measured between 4 and 25.

### 3.4 Validation with Fluorescent Beads in 3D Hydrogel

**Fig. 8:**
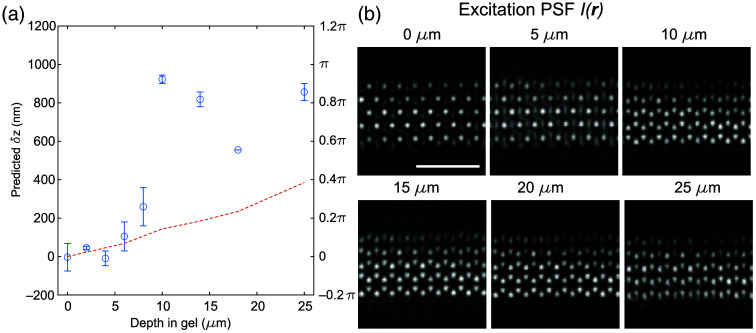
Demonstration of method in 3D hydrogels. (a) Comparison between the experimentally measured (red dashed lines) and computationally predicted (blue circles) axial offset between the excitation and detection focal plane in images of fluorescent beads taken at different depths in a 3D polyacrylamide hydrogel. In this case, no offset was experimentally applied, but the hydrogel introduced a shift in the excitation pattern that varies as a function of depth. (b) Experimentally measured excitation pattern (I(r)) taken at different depths in the hydrogel. Scale bar=5  μm.

Thus far, we have validated that our method can posteriorly extract the axial offset of the excitation pattern from raw SIM images of beads and adherent cells. However, these structures are still relatively thin. To address how our algorithm would perform in thicker samples, we next tested its performance using fluorescent beads embedded within thick 3D hydrogels. Because beads in the imaged hydrogels span multiple locations along the light-sheet propagation direction and to exclude possible aberrations from this aspect (covered in Fig. 6), we only include beads illuminated by ±5  μm within the focus along the light-sheet propagation direction when computing the axial offset of the beam. To quantitatively validate whether the prediction from our methodology is correct and assess additional artifacts from imaging through the hydrogel, we also independently measured the offset between the excitation focus (determined by the axial profile of the lattice light sheet) and the detection focus (determined by the axial profile of the bead illuminated by widefield illumination). As shown in Fig. 8(a), our method can correctly extract the sample-induced excitation axial offset within 5  μm below the hydrogel surface. However, our method tends to overestimate this offset for beads more than 10  μm below the hydrogel surface. This mismatch is likely attributed to the distortion of the illumination pattern (and possibly the detection PSF) caused by the hydrogel refractive index mismatch with the media [Fig. 8(b)]. The excitation aberration becomes visually apparent when the depth is larger than 10  μm. Therefore, the assumption of our method that, other than axial or lateral translations, the same excitation pattern and detection PSFs are applied to take both the transfer function library ${{\overset{˜}{O}}^{\prime }}_{m}(k)$ and the sample images $\overset{˜}{D}(k)$ is no longer valid.

## 4 Discussion

In this paper, we introduce a posterior method for extracting the axial offset between the excitation light sheet and the detection focal plane in latticeSIM. We validated this method through simulations and experiments, employing fluorescent beads, adherent cells, and 3D hydrogels. Our demonstrations illustrate that identifying the transfer functions with the same retrieved axial offset as that of the raw SIM image minimizes artifacts in the reconstructed images. Overall, this approach enables posterior correction of system or sample-induced axial offsets in latticeSIM. Importantly, it only necessitates a gallery of transfer functions with varying axial offsets that can be acquired experimentally or computationally generated, without the need for additional optical components in the microscope.

One caveat of our method is that with decreasing SNR, it can become challenging to differentiate between the axial focal position of the light sheet and the position that is half a period off [see Figs. 5(a) and 6(a)]. This challenge is particularly pronounced for hexagonal lattice patterns with a small difference between the minimum and maximum NA of the bounding pupil mask. The x-averaged axial intensity profile shown in Figs. 1(c) and 6(c) inset reveals that the center peak (representing the true axial focal position) and the two side lobes (representing axial positions that are half a period off) possess similar magnitudes. Consequently, this explains why images reconstructed with the correct axial offset or those shifted by half a period exhibit comparable image qualities. We expect that lattice patterns with more pronounced differences in intensity between the center and side lobes (e.g., a square lattice or a hexagonal lattice with a larger NA gap) will exhibit greater disparities between the two minima corresponding to the center peak and the side lobes, making them easier to distinguish via our approach, but also more sensitive to artifacts when it fails and inaccurately identifies a half-period shifted pattern.

Another limitation of our approach is that it assumes that the same illumination pattern is applied in both the SIM image and during the calibration of the transfer function library. This necessitates that, other than translational shifts, the OTFs of the excitation pattern and detection objective remain identical between the two instances; failure to meet this assumption can result in artifacts during reconstruction. For example, alterations in the magnitude distribution within the excitation OTF relative to that of the transfer function library will lead to incorrect normalization during SIM reconstruction and introduce artifacts. Furthermore, sample-induced aberrations that cause distortion of the lattice pattern will result in erroneous predictions by our method, as demonstrated in the 3D hydrogel where aberrations induced by a refractive index mismatch distorted the hexagonal illumination pattern. Finally, even in systems with adaptive optics, correction for aberrations across the entire biological sample can be challenging. Thus, a single posterior correction may not be valid over an entire image. In these cases, we postulate that our method could be combined with tiled SIM reconstruction^17^ to account for this spatially variant offset.

We demonstrated that our method can correct for axial offset over the range of over 2.5  μm (slightly over the full-width half max of the beam’s axial Gaussian envelope). In practice, the range of recoverable axial offset will depend on the range of calibration PSF library being collected, the bounding envelope of the light sheet, and the axial period of the lattice illumination. For an offset of more than one axial period of the lattice, our approach may have trouble disambiguating between the true optimal position at the center of the light-sheet envelope and those that are located either one-half or one period away as described above [e.g., the flanking minima in Figs. 5(b) and 5(c)]. When sample-induced phase aberrations are present, we illustrate that our method cannot correct for the aberration caused by hydrogel samples with thicknesses over 7  μm; however, this will be dependent on the refractive index mismatch between the sample and immersion media.

Nevertheless, we demonstrate here that the method performs well for adherent cells where the light-sheet offset can be considered constant across the usable field of view. As these examples have made up the majority of use cases for latticeSIM, we envision that our method will be a valuable addition to restoring image quality even under non-ideal experimental conditions. Furthermore, if we view the axial offset of the excitation beam as a phase aberration (e.g., Noll Zernike indices 2 and 3), then we envision that extensions of this method may be used to correct for higher-order or even arbitrary phase aberrations in the excitation beam as long as they can be represented in a gallery of experimentally obtained or simulated transfer functions and used for residual phase minimization. This advancement would further enable tiled reconstruction for latticeSIM experiments in complex 3D specimens.

## Supplementary Material



## Data Availability

Due to the inordinate size of the image data (∼700  GB), it is not currently feasible to deposit this into a central repository; however, all datasets underlying the results in this paper are available from the corresponding author upon request. To the extent possible, the authors will try to meet all requests for data sharing within 2 weeks from the original request. Code to reproduce the results shown in this paper, a small demonstration dataset, and the setting file for SIM reconstruction are available at: https://github.com/legantlab/SIM_Aligment.
