# Supplementary material for: Posterior approach to correct for focal plane offsets in lattice light-sheet structured illumination microscopy
Source: J Biomed Opt. 2024 Jul 31;29(8):086502. doi: 10.1117/1.JBO.29.8.086502 (PMC11289499; doi:10.1117/1.JBO.29.8.086502)
Supplement: Supplementary file 1 [file JBO_029_086502_SD001.pdf]

Table of Contents

Supplementary Note 1	2-3
Supplementary figures	4

Supplementary Note 1

Comparing Lattice SIM and I5S

Our method for detecting the axial offset between the detection and excitation focal planes is inspired by an earlier work which introduced an approach to correct for path length differences in the interferometric arms of an I5S microscope (Shao et al 2012 [14]). Both our method and Shao et. al. leverage residual phase information in the overlap regions between different lateral information components in $\tilde{J}_m(k_x, k_y)$. Here, we review the similarities and differences between our approach and that derived previously for I5S.

I5S microscopy obtains ~100 nm isotropic resolution by combining the lateral resolution enhancement of 3-beam structured illumination microscopy with interferometric excitation and detection through two axially opposed objective lenses, similar to 4PI or IM microscopy [18]. One of the major challenges for these microscopes is to maintain equal pathlength between the two arms of the interferometer. Deviations from this condition result in an axial shift of the interference fringes of detection PSF. In frequency space, this shift manifests as phase offsets in the interferometric side bands of the detection OTF. In I5S, the excitation pattern is formed by the coherent interference of 6 beams, three beams from each of the two opposing objective lenses. Together, these six beams form a single pupil function that is very similar to the pupil function for latticeSIM with a hexagonal lattice pattern. In I5S, the excitation path is shared with the detection path, thus the same interferometer path length differences that lead to shifts in the detection PSF fringes also lead to axial shifts in the excitation pattern relative to the mutual focal plane of the two objective lenses. Shao et. al. 2011 demonstrated that the path length difference can be detected by comparing the phase differences in the overlap regions of the laterally separated information components. Initially, this process is complicated by the interferometric detection OTF sidebands which also acquire a path length-dependent phase shift. However, the detection OTF sidebands can be made to cancel out by registering and summing images from both the primary and secondary exits of the 50-50 beam splitter at the exit of the interferometer. In this manner, the separated information components now consist of a standard (non-interferometric) detection OTF that has acquired the phase components of the axially shifted excitation pattern. In Shao et al. 2011, the authors approximated the six focused spots in the excitation pupil function of I5S as Dirac delta functions and then derive an analytical solution for the residual phase in the overlap region that is similar to Equation 13 in this work.

While our approach relies on a similar conceptual framework, there are a few important differences to consider when adapting this method to latticeSIM. First, and most apparent is that there is no need to deal with the interferometric side bands of the detection OTF

which simplifies the analysis. However, in contrast to I5S, it is generally not feasible to approximate the excitation pupil function as a superposition of delta functions due to the axial confinement envelop of the light sheet. More specifically, each of the 19 frequency components of the excitation OTF take the form of elongated lines in k_z. When there is an offset between the light sheet and the detection focal plane, each of these lines acquire a linear phase gradient rather than a single scalar value. This complicates the analytical solution and due to these factors, we favor solving for the minimal residual phase using a library of experimentally obtained PSFs. Finally, the I5S approach does not have to deal with potential misalignment along the propagation direction of the light sheet. In essence, this introduces a defocus phase onto the excitation pupil function that can potentially affect the analysis as we demonstrate in Figure 6 of this manuscript.

Supplementary References:

14. Shao, L., Winoto, L., Agard, D. a., Gustafsson, M. g. l. & Sedat, J. w. Interferometer-based structured-illumination microscopy utilizing complementary phase relationship through constructive and destructive image detection by two cameras. *Journal of Microscopy* **246**, 229–236 (2012).

18. Bewersdorf, J., Schmidt, R. & Hell, S. W. Comparison of I5M and 4Pi-microscopy. *J Microsc* **222**, 105–117 (2006).

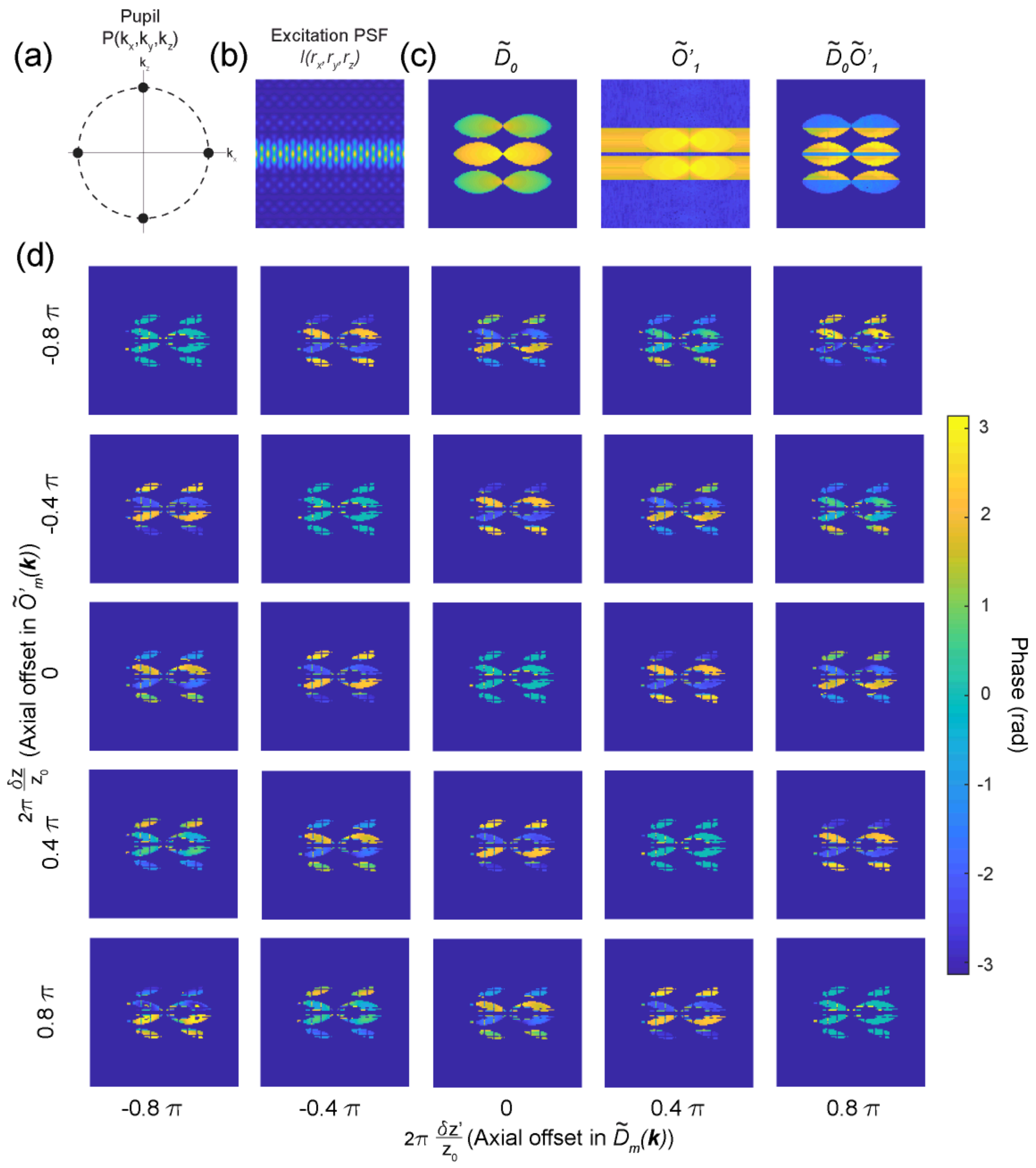

Figure S1 Residual phase in OTF for square lattice. (a) The excitation pattern at the back pupil plane of the excitation objective for a square lattice pattern. **(b)** The excitation point spread function (PSF) of the square lattice pattern (min and max NA is 0.5 and 0.55 respectively) that illuminates the sample. **(c)** Magnitude of the zeroth information component ($\tilde{D}_0$), the first SIM transfer function ($\tilde{O}'_1$) after being shifted to the correct position in frequency space, and the magnitude of the product of the two for square lattice. This panel follows the same convention as **Fig 3(a)**. **(d)** Residual phase for square lattice pattern as computed via eqn (12) for different axial offsets between the excitation and detection planes in simulated SIM images (different columns) and simulated transfer functions (different rows). This panel follows the same convention as **Fig 3(d)**.
